# Leprosy in children under fifteen years of age in the most hyperendemic municipality in Brazil

**DOI:** 10.1590/1984-0462/2024/42/2023022

**Published:** 2023-10-23

**Authors:** Ricardo Milhomem Costa, Marcela Silva Menezes, Maria Sortênia Alves Guimarães, Eliane Patrícia Pereira Franchi, Lorena Dias Monteiro, Mariana Caroline Tocantins Alvim

**Affiliations:** a Universidade Federal do Tocantins, Palmas, TO, Brazil.

**Keywords:** Leprosy, Neglected diseases, Epidemiology, Children, Hanseníase, Doenças negligenciadas, Epidemiologia, Crianças

## Abstract

**Objective:**

To describe leprosy involvement and physical disability profiles in children and adolescents under 15 years old.

**Methods:**

Ecological time series study, based on data from the Brazilian Notifiable Diseases Information System, including new cases of leprosy residing in Palmas (TO), from 2001 to 2020.

**Results:**

A total of 471 notified cases in children and adolescents under 15 years of age were evaluated, resulting in a detection coefficient of 26.5 per 100,000 inhabitants. Of these, 52% (n=243) were women, 5% (n=24) corresponded to grade two disability, and 36% (n=168) were diagnosed through spontaneous demand. The temporal trend analysis showed a 0.5% reduction in the detection coefficient. There was a significant decrease in the diagnosis of the undetermined and tuberculoid clinical forms and a significant increase in the dimorphous form. Diagnosis through contact examination increased significantly by 13.1% and that through spontaneous demand decreased by 4.9%. The detection coefficient of cases with grade two disability reduced significantly by 7.4% while those with grade one increased by 16.8%.

**Conclusions:**

Despite the downward trend in the detection coefficient in children and adolescents under 15 years of age and in cases with grade two disability, other factors indicate failure in the adequate management of leprosy in Palmas.

## RESUMO

**Objetivo:**

Descrever os perfis de acometimento de hanseníase e incapacidade física em menores de 15 anos.

**Métodos:**

Estudo ecológico de série temporal, baseado em dados do Sistema Nacional de Agravos de Notificação, incluindo casos novos de hanseníase residentes em Palmas (TO), no período de 2001 a 2020.

**Resultados:**

Foram avaliados 471 casos notificados em crianças e adolescentes menores de 15 anos, resultando em um coeficiente de detecção de 26,5 por cem mil habitantes. Destes, 52% (n=243) eram do gênero feminino, 5% (n=24) correspondiam ao grau dois de incapacidade física, e 36% (n=168) foram diagnosticados por demanda espontânea. A análise de tendência temporal mostrou queda do coeficiente de detecção em 0,5%. Houve queda significativa no diagnóstico das formas clínicas indeterminada e tuberculoide e aumento significativo da dimorfa. O diagnóstico por exame de contato teve um aumento significativo de 13,1% e o por demanda espontânea, queda significativa de 4,9%. O coeficiente de detecção de casos com grau dois de incapacidade apresentou uma queda significativa de 7,4%, enquanto o de casos com grau um, apresentou um aumento de 16,8%.

**Conclusões:**

Apesar da tendência de queda do coeficiente de detecção em menores de 15 anos e do coeficiente de detecção de casos com grau dois de incapacidade, outros fatores indicam falha no manejo adequado da hanseníase em Palmas.

## INTRODUCTION

Leprosy is an infectious disease transmitted through intimate and prolonged contact, as well as through the airways. The etiological agent is *Mycobacterium leprae (M. leprae)*, an acid-fast, weakly gram-positive bacillus that infects peripheral nerves, specifically the Schwann cells of superficial nerves of the skin and peripheral nerve trunks, thus affecting the eyes and organs.^1^ It is an endemic pathology in several regions worldwide, including Brazil, in which there are more than 90% of cases in the Americas.^1,2^

The symptoms and signs of leprosy include changes in skin sensitivity, papules, nodules, and thinning hair. Other symptoms include pain, thickening of peripheral nerves, muscle weakness, swelling of hands and feet, fever, joint pain, and dryness in the nose and eyes. Symptoms are related to the organism's reaction to *M. leprae*. The disease presents the forms: undetermined (paucibacillary), characterized by a single skin lesion; tuberculoid (paucibacillary), resulting in an anesthetic plaque or nodule or thickened nerve with total loss of sensitivity; borderline (multibacillary), presenting several lesions with decreased sensitivity and autonomic functions; and lepromatous, a highly contagious form causing skin infiltration, dryness, and dilated pores.^1,3^

Treatment stops bacterial transmission and cures the disease. Without proper treatment, Hansen's disease can become transmissible and lead to physical disabilities and incapacities, such as ulnar nerve injury causing fixed finger flexion, or the loss of thumb opposition due to median nerve injury. Radial nerve injury that hinders wrist extension may also occur, as well as, posterior tibial trunk injury that can cause toe clawing and loss of sensation in the plantar region, and common fibular nerve injury that leads to gait alteration and inability to lift the foot. In addition, facial nerve injury can also happen, resulting in the inability to close the eyelids.^1–3^

Due to the long incubation period of the disease (from two to seven years on average), the occurrence of cases in children and adolescents aged under 15 years indicates active transmission foci, an important indicator for monitoring the endemic disease.^4,5^ Between 2010 and 2019, a total of 20,684 new cases of leprosy in children under 15 years of age were diagnosed in Brazil, with a reduction in the detection rate of 55.2%, from 5.34 per 100,000 inhabitants in 2010 to 3.44 in 2019, meaning a change in the parameter from “very high” to “high”. Overall, 23,612 new cases of leprosy were diagnosed in Brazil in 2019, of which 1319 (5.6%) occurred in children under 15 years of age through referral and spontaneous demand in 82% of cases.^6^ Palmas (TO) detected a general rate of 271,37 cases per 100,000 inhabitants in 2018 and 226,99 in 2019, the highest in the country, considering only the capitals.^6–8^

Although there is a trend towards a reduction in cases of leprosy, factors persist that make services insufficient. This situation is even worse when the index case is male, black, and residing in the rural area, which reinforces aspects of social vulnerability and the need for increasing the effectiveness of control actions.^9^

Considering the severity of the disease in Palmas (TO) — the most hyperendemic capital for leprosy in Brazil — and the low performance of activities for active search, leprosy management, and social vulnerability,^4,7,10^ the present study aimed at describing the profiles of leprosy involvement and physical disability in children and adolescents under 15 years of age, who were treated at a healthcare service in the city of Palmas (TO), between the years 2001 and 2020.

## METHOD

This is an ecological time series study, based on data from the Brazilian Notifiable Diseases Information System (SINAN), including new cases of leprosy living in Palmas (TO), from 2001 to 2020.

The analyzed unit was Palmas, a north Brazilian municipality with an area of 2,227,444 km^2^, the capital and largest city in Tocantins. The population reached 313,349 inhabitants after 30 years of its creation, with a growth rate of 2.4%, making it the least populous capital in Brazil.^11^ The Family Health Strategy (ESF) has covered 100% of the population since July 2016.^12^

Data from notifications of leprosy cases in children and adolescents under 15 years of age were included, and those referred to as residing in other municipalities were excluded. For descriptive analyses, variables were selected based on annual case records. The selected indicators were those recommended by the National Program for Leprosy Control: detection coefficient in children under 15 years of age (indicating active transmission of the disease); multibacillary cases (for late diagnosis); paucibacillary cases proportion (indicating early diagnosis); detection mode (for the analysis of service's diagnostic capacity) and the proportion of all new cases detected during the year for new cases with grades one and two of disability (used to assess late diagnosis as an indicator of the quality of search activities for cases).^13^

Physical disabilities and functional loss were categorized into three grades based on the Simplified Neurological Assessment: Grade 0 (no neural impairment), Grade 1 (decreased sensitivity or muscle strength), and Grade 2 (visible physical deformities).^14^

Data were obtained from notifications stored in SINAN, from the Ministry of Health. This system is filled by case notification and investigation forms, and it allows the identification of events of public health relevance, providing subsidies for diagnosing the epidemiological situation of a given region.^15^ Population data were collected from the Brazilian Institute of Geography and Statistics (IBGE), based on the 2010 Census and population estimates for the intercensal years 2001–2009 and 2011–2020.^11^

Analyzes of temporal trends of leprosy indicators for the study period were performed using the Poisson joinpoint regression model (by inflection points). The year of occurrence was considered as an independent variable, and the leprosy indicators in children under 15 years of age for Palmas, as dependent variables.

The joinpoint model detected trends and changes in indicators, allowing the calculation of the Annual Percent Change (APC) and weighted geometric Average Annual Percent Change (AAPC) at a 95% confidence interval (95%CI) and significance level of 5%. This model tested whether a multisegmented line was statistically better at describing the temporal evolution of a data set than a straight or less segmented line.^16^ Joinpoint Regression Program 4.9.0 analyzed the data, while Microsoft Excel generated tables and graphs.

This study is part of the project Epidemiological and Health Service Standards Related to Low-Quality Assessment of Leprosy Contacts in the Health Care Network of Palmas, Tocantins, and received approval from the Research Ethics Committee of the Lutheran University Center of Palmas, under protocol 3.251.050 and CAAE 79187717.7.0000.5516.

## RESULTS

The study analyzed all leprosy cases in children and adolescents under 15 years of age reported in Palmas (TO) from 2001 to 2020, totaling 524 cases. After applying exclusion criteria for cases from other municipalities or with different modes of entry, 471 were included in the analysis, resulting in a detection coefficient of 26.5 per 100,000 inhabitants. Of these, 52% (n=243) were female, 5.0% (n=24) had grade two disability, and 36% (n=168) were diagnosed through spontaneous demand (Table 1).

**Table 1:** Epidemiological and operational indicators of leprosy in children under 15 years old living in Palmas (TO), 2001–2020.

	DC*	% Male†	% G0‡	% G1§	% G2//	% Pauc.¶	% Multib.#	% Ref.**	% Sd.††	% Col.‡‡	%Cont.§§
2001	47.4	58	91.7	0	8.3	91.7	8.3	20.8	66.7	0	12.5
2002	35.6	37	94.7	0	5.3	68.4	31.6	31.6	63.2	0	5.3
2003	46.2	54	69.2	3.9	26.9	84.6	15.4	23.1	50	7.7	7.7
2004	25.4	47	93.3	0	6.7	80	20	20	60	6.7	13.3
2005	27.5	29	70.6	11.8	17.7	88.2	11.8	35.3	52.9	0	11.8
2006	22.4	50	85.7	14.3	0	71.4	28.6	35.7	50	0	7.1
2007	28.6	50	83.3	11.1	5.6	66.7	33.3	27.8	50	0	16.7
2008	20.6	69	92.3	0	7.7	84.6	15.4	15.4	69.2	0	15.4
2009	25.2	63	87.5	12.5	0	75	25	18.8	62.5	0	12.5
2010	20.5	38	61.5	30.8	7.7	46.2	53.9	30.8	61.5	0	7.7
2011	15.4	40	100	0	0	70	30	20	50	0	30
2012	12.1	38	100	0	0	87.5	12.5	37.5	50	0	12.5
2013	19.2	31	61.5	30.8	7.7	46.2	53.9	15.4	61.5	0	15.4
2014	18.8	46	100	0	0	92.3	7.7	38.5	38.5	7.7	15.4
2015	22.7	56	87.5	12.5	0	37.5	62.5	31.3	18.8	43.8	6.3
2016	73	48	63.5	30.8	5.8	21.2	78.9	5.8	23.1	25	46.2
2017	62.6	60	64.4	28.9	6.7	8.9	91.1	17.8	13.3	4.4	57.8
2018	79.6	48	72.4	27.6	0	1.7	98.3	8.6	13.8	8.6	55.2
2019	74.5	47	78.2	21.8	0	3.6	96.4	14.5	20	0	63.6
2020	34.8	35	61.5	38.5	0	0	100	7.7	15.4	0	73.1

* Detection coefficient <15 years per 100,000 inhabitants;

† % Male;

‡ % Grade zero;

§ % grade one;

// % Grade two;

¶ % Paucibacillary;

# % Multibacillary;

** % Diagnosis by referral;

†† % Diagnosis by spontaneous demand;

‡‡ % Diagnosis by collective examination;

§§ % Diagnosis by contact tracing.

**Figure 1:**
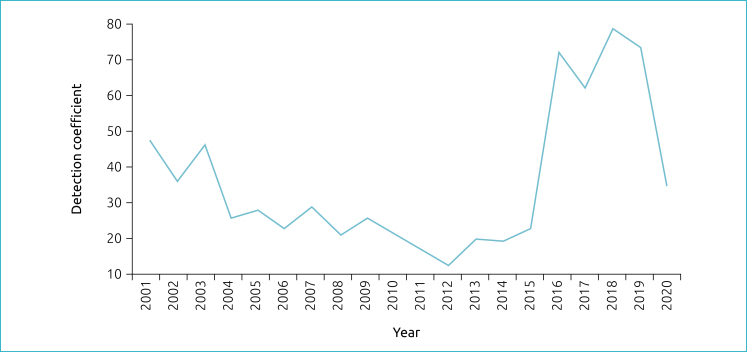
Trend in detection coefficient of new cases of leprosy in children under 15 years of age per 100,000 inhabitants, in Palmas (TO), 2001–2020.

In the temporal trend analysis, the detection coefficient in children under 15 years of age presented a statistically significant reduction of 9.9% between 2001 and 2012, followed by a considerable increase of 36.4% from 2012 to 2018, and a non-significant decrease of 32.9% between 2018 and 2020. In the total period, there was a non-significant decrease of 0.5% (Figure 1). Between 2019 and 2020, the first year of the COVID-19 pandemic, the coefficient dropped from 74.52 to 34.80, a reduction of 53.3% (Table 1).

There was a noticeable decrease in the diagnosis of the undetermined and tuberculoid forms, a non-significant decrease in the lepromatous form, and a significant increase in the borderline form (Table 2). Diagnosis by examination of contacts reached an elevated increase of 13.1% between 2001 and 2020, while cases detected by collective examination were not substantial in the same period, even with the considerable drop between 2015 and 2020 (Table 3). There was a significant drop of 4.9% of cases diagnosed by spontaneous demand between 2001 and 2020 and a non-significant drop of 1.3% of cases detected by referral (Table 3). The detection coefficient of cases with grade two disability presented a significant decrease of 7.4% in the total period, while those with grade one increased by 16.8% (Table 2).

**Table 2:** Trend of epidemiological indicators of leprosy in children under 15 years of age in Palmas (TO) according to joinpoint regression analysis, 2001–2020.

		Detection coefficient*	% Paucibacillary	% Multibacillary	% Undetermined	% Tuberculoid	% Borderline	% Lepromatous
Trend 1	Period	2001/12	2001/20	2001/12	2001/20	2001/20	2001/20	2001/13
	APC	-9.9†	-9.4†	-0.7	-9.6†	-11.1†	20.4†	-3.2
	95%CI	-15.3; −4.2	-12.3; −6.3	-13.9; 14.4	-12.6; −6.5	-15.0; −7.1	13.4; 27.8	-10.8; 5.1
Trend 2	Period	2012/18		2012/18				2013/17
	APC	36.4*		55.0*				34.9
	95%CI	14.5; 62.4		18.0; 103.6				-12.4; 107.6
Trend 3	Period	2018/20		2018/20				2017/20
	APC	-32.9		-33.7				-44.4
	95%CI	-63.8; 24.4		-67.3; 34.4				-69.7; 1.8
	AAPC	-0.5	-9.4*	9.5	-9.6*	-11.1*	20.4*	-4.9
	95%CI	-8.4; 8.2	-18.6	-3.5; 24.3	-12.6; −6.5	-15.0; −7.1	13.4; 27.8	-16.3; 8.0

APC: Annual Percent Change; AAPC: Average Annual Percent Change; 95%CI: 95% confidence interval.

* Detection coefficient < 15 years/100,000 inhabitants;

† Significantly different from 0 (p<0,005).

**Table 3:** Trend of operational indicators of leprosy in children under 15 years of age in Palmas (TO), according to joinpoint regression analysis, 2001–2020.

		% G0*	% G1†	% G2‡	% G2c§	% Cont.//	% Col.¶	% Ref.#	% Sd.**
Trend 1	Period	2001/13	2001/20	2001/20	2001/20	2001/20	2001/12	2001/20	2001/20
	APC	-8.8††	16.8††	-7.4††	-9.0††	13.1††	-4.7	-1.3	-4.9††
	95%CI	-12.4; −4.9	10.1; 23.9	-13.5; −0.7	-13.5; −4.4	10.2; 16.0	-19.6; 13.0	-4.4; 2.0	-7.5; −2.2
Trend 2	Period	2013/18					2012/15		
	APC	40.5††					138.1		
	95%CI	16.8; 69.0					-78.2; 2.498.1		
Trend 3	Period	2018/20					2015/20		
	APC	-33.1					-47.2††		
	95%CI	-60.4; 13.0					-60.0; −30.3		
	AAPC	-1.1	16.8††	-7.4††	-9.0††	13.1††	-5.7	-1.3	-4.9††
	95%CI	-7.8; 6.1	10.1; 23.9	-13.5; −0.7	-13.5; −4.4	10.2; 16.0	-34.0; 34.8	-4.4; 2.0	-7.5; −2.2

APC: Annual Percent Change; AAPC: Average Annual Percent Change; 95%CI: 95% confidence interval.

* % Disability Grade zero;

† % Disability Grade one;

‡ % Disability Grade two;

§ Disability Grade two/100,000 inhabitants;

// % Cases detected by contact tracing;

¶ % Cases detected by collective examination;

# % Cases detected by referral;

** % Cases detected by spontaneous demand;

†† Significantly different from 0 (p<0,005).

**Figure 2:**
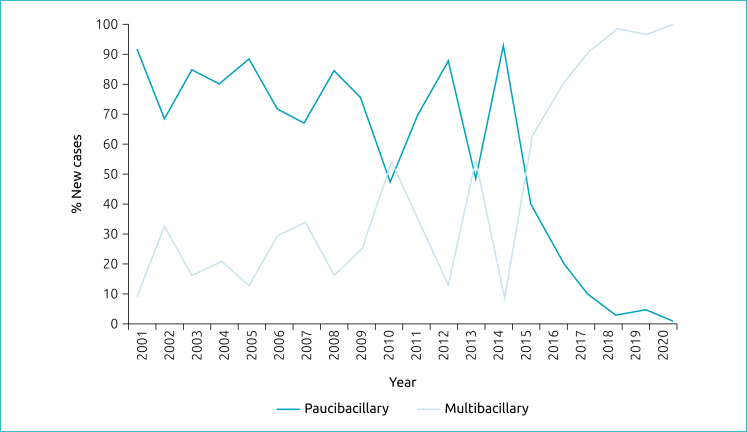
Percentage of new cases of leprosy in children under 15 years of age residing in Palmas (TO) according to operational classification, 2001-2020.

Regarding the percentage of new cases of leprosy in children under 15 years of age according to the operational classification (Figure 2), there was a hegemony of cases classified as paucibacillary until 2010; after that, there was a small predominance of cases classified as multibacillary. The primacy of cases classified as multibacillary repeated in 2013 and it was only fixed from 2015 onwards.

In 2001, 2003, 2008, 2009, 2015, and 2017, there was a predominance of male cases, and in 2006 and 2007, a balance between the two genders was observed (Table 1).

## DISCUSSION

The results obtained in this study reveal periods of “high” and “very high” disease burden, and hyperendemic parameters, which are similar to the results obtained by Monteiro et al.,^17^ between 2001 and 2016. These results showed a considerable reduction in the number of newly diagnosed cases in children under 15 years of age.^5,17^

This situation represents the expression of inequalities and social vulnerabilities in a city that, even being young, reproduces the exclusion model characteristic of Brazil and it differs from the sudden drop in the detection coefficient in children and adolescents, from 2008, in Tocantins.^18^ Three periods of trend in the detection coefficient in children under 15 years of age can be explained by the apparent operational disorganization of the health services in force before the creation of the program “Palmas Livre da Hanseníase” (Palmas Leprosy-free), which culminated in a considerable upward trend from 2012, in a peak in 2018, and in a non-significant reduction thereafter (Table 2). As mentioned by Monteiro et al.,^18^ the undiagnosed prevalence can reach eight times that of the registered cases, increasing the infection risk in the population.^18^

The diagnosis in children is difficult.^19^ Clinical signs may not be recognized in childhood due to the difficulty in applying and interpreting sensitivity tests in this age group. However, any health professional should be aware of the diagnostic suspicion in contacts of patients with a history of the disease, especially in hyperendemic areas, since the child population comes into early contact with the bacilliferous patient in these areas. Aptitude for judicious examination of cases is also required.^20^

Evaluating the entire study period, the predominance of new cases detected by spontaneous demand (36%) and contact exams (36%) differs from that found in Goiânia (GO) by Nunes et al.,^21^ and in Fortaleza (CE) by Alencar et al.^19^ In those studies, 54.05% and 75% of the cases, respectively, were detected by referral, meaning that the surveillance of individuals with leprosy should be carried out through detection in the population present in the health services or referrals in the health care network.^19,21^ This passive case detection is strongly related to the operational fragility of leprosy surveillance.^18^ Also, finding a low proportion of cases diagnosed by referral or examinations by the community may mean a failure in the attention of professionals at health care units in relation to the early disease diagnosis, even with a predominance of grade zero of physical disability observed in the present study. The professionals themselves are often unaware of the main signs and symptoms of leprosy, favoring physical disabilities as sequelae.^22^

Still, the significant increase in detection by examination of contacts and drop by spontaneous demand suggests that, when leprosy is in focus in the evaluation, suspicion becomes more of a diagnosis. This result differs from that obtained by Monteiro et al.,^18^ who considered the diagnosis by evaluation of contacts precarious, with an average of 17.6%, and pointed out the strong correlation between illness in children and active bacilli foci in the family environment.^18^

Since 2018, there has been an increase in the detection rates of new cases of leprosy in children under 15 years of age and in patients with grade two disability per 100,000 inhabitants in Palmas (TO) (Table 1). This fact may be related to the poor quality of leprosy management with consequent late diagnosis and a high rate of cases being detected.^6^

The COVID-19 pandemic, caused by the SARS-CoV-2 virus or the New Coronavirus, has impacted all fields of human activity. In more acute pathologies such as meningitis and COVID-19, non-pharmacological measures to reduce transmissions, such as social isolation, reduction in the number of consultations, and absence of health professionals (infected by the virus), in addition to the fear of the population being assisted in hospitals and health centers, may have influenced the lower number of notifications in 2020.^23^

In the case of leprosy, the decrease in the number of records in 2020 can be justified by the adoption of isolation and social distancing measures during the pandemic.^10,24^ Consequently, leprosy underreporting may have been aggravated from this period onwards since it is a pathology whose incubation period can reach ten years and the domestic environment is a place of great transmission.

Between 2019 and 2020, the first year of the COVID-19 pandemic, the detection coefficient in children under 15 years old in Palmas ranged from 74.52 to 34.80, a reduction of 53.3% (Table 1). This reduction is according to the results found by Cunha et al.,^24^ who estimated 177% of underreporting cases in 2020.^24^

Considering the average number of new cases between 2010 and 2019, compared to 2020, there was a consistent reduction in all Brazilian regions, ranging from 41% in the Midwest to 56.4% in the Southeast. In Brazil as a whole, the number of cases decreased by 48.4%.^25^

The detection coefficient by contacts evaluation indicates the operational capacity of the services to diagnose. Until 2014, this proportion represented 6.3% of detections in Palmas (Table 1). Since the creation of the program “Palmas Livre da Hanseníase”, this proportion has increased by 201.1%, demonstrating an improvement in the operational capacity. However, the adequacy of the detection coefficient by evaluation of contacts suffers losses due to the problems faced in the management of leprosy, such as the turnover of professionals, lack of supplies, lack of adequate application of protocols (training, adverse reactions), taboos in relation to the disease and its insidious characteristic.^11,26,27^

Palmas has full coverage of ESF teams, which, in theory, favors proper follow-up of contacts and early diagnosis, reducing disability at the time of diagnosis. For Monteiro et al.,^17^ the examination of household contacts determines the lowest risk of late diagnosis. It highlights the importance of this mandatory measure in leprosy control programs in Brazil.^17^

Multibacillary forms are more common in adults than in children due to their immature immunity. The greater general prevalence of multibacillary cases and the upward trend (Figure 2) found here are of concern, as they reflect a tendency towards difficult healing in many patients and differ from many authors, such as Corpes et al.,^28^ who found a predominance of paucibacillary cases in their studies.^28^

As a condition that not only prevails in situations of poverty but also contributes to the perpetuation of inequalities, the characterization of leprosy as a neglected disease becomes evident when one examines the low percentage of the undetermined clinical form in relation to the borderline form, despite the latter being more closely associated, along with tuberculoid.^29^

The prevalence of the disease in females (52%) identified in the present study differs from that found by many authors, especially by Corpes et al.^28^ The disparity can be justified by the female cultural characteristic of being more attentive to her own health and the organization logic of health services.^29^

Because leprosy is a disease with a characteristic of prolonged progression, the degree of disability is related to the duration of the disease and it allows an indirect assessment of both operational and epidemiological components since late diagnosis favors disease foci perpetuation. The predominance of cases with grade zero disability is related to the short time taken to diagnose the disease since contamination. Even so, the significant increase in the detection of new cases with grade one, even with a reduction in grade two, may mean a decrease in the effectiveness of early detection actions, resulting in multibacillary cases, reactional episodes, and physical disabilities (Table 1). Physical deformities at the time of diagnosis indicate the disease severity.^18^

The pillars of leprosy control include early diagnosis, correct treatment, monitoring of reaction signs, immediate treatment of reactions, examination of contacts, and vaccination. The strengthening of surveillance, thus, includes public health actions with an active search for primary sources of infection and prevention of new cases in childhood, adding social contacts, campaigns, and educational actions within the most endemic areas, aiming at early diagnosis and treatment.^5^

In conclusion, the present study emphasizes that, despite the downward trend in the detection coefficient in children under 15 years of age and cases with grade two of disability during the evaluated period, the predominance of multibacillary cases since 2015 and new cases detected through spontaneous demand, along with the continued high disease burden, may indicate a failure in leprosy management, suggesting active transmission, delayed diagnosis, and highlighting its neglected disease status. The possibility of a hidden prevalence of leprosy persisting due to limited patient access to health services resulting from the COVID-19 emergence should not be disregarded, and upcoming studies should evaluate longer periods of the pandemic crisis. Finally, the interpretation of the results must consider the limitations of studies based on secondary data. Nevertheless, the findings of this study may be useful in the public health context to underscore the need for reassessing leprosy control strategies by improving health services and surveillance to ensure mandatory diagnoses and notifications and to support further studies.

## Data Availability

The database that originated the article is available with the corresponding author.
